# Use of and attitudes towards the prescribing guidelines booklet in primary health care doctors

**DOI:** 10.1186/1472-6904-8-8

**Published:** 2008-09-22

**Authors:** Magnus AB Axelsson, Malin Spetz, Anders Mellén, Susanna M Wallerstedt

**Affiliations:** 1 Department of Clinical Pharmacology, Sahlgrenska University Hospital, SE-413 45 Göteborg, Sweden

## Abstract

**Background:**

In the region of Västra Götaland in Sweden, prescribing guidelines, drawn up by 24 expert groups and determined by the regional board for drugs, are since 2006 available in the form of an annually published booklet. This study investigates, for the first time, the use of and attitudes towards this publication.

**Methods:**

A questionnaire was administered to doctors working in primary health care in the region of Västra Götaland in Sweden. Questions included characteristics of the responding doctor and use of the prescribing guidelines booklet, as well as attitude questions constructed as statements to which the responder should grade his level of agreement from 1 (total disagreement) to 6 (total agreement).

**Results:**

Totally 603 filled-in questionnaires were returned (estimated response rate 60%). The majority of the doctors (n = 571, 97%) responded that they use the prescribing guidelines booklet, and when prescribing a drug for a new diagnosis, a drug from the booklet is chosen in most cases [median (25^th ^– 75^th ^percentile) 80 (75–90)]. However, at renewal of a drug prescription, active change to a drug from the prescribing guidelines booklet occurs less often [median (25^th ^– 75^th ^percentile) 50 (20–70)]. The booklet also includes short therapy advice sections, which 231 doctors (42%) use every day and 191 (34%) use every week. The attitudes towards the prescribing guidelines booklet were generally positive. Doctors in privately run primary health care units and doctors running their own business were generally more negative and judged themselves to be less adherent to the prescribing guidelines booklet compared with doctors in publicly run primary health care units.

**Conclusion:**

The prescribing guidelines booklet is frequently used and is generally appreciated, though differences exist between subgroups of users.

## Background

Drugs are one of the keystones in the treatment of patients. Rational use of drugs requires adequate knowledge on benefits, risks and cost-effectiveness of drugs. Knowledge in the drug area is rapidly increasing and it may be difficult for a primary care doctor to be updated in all therapeutic areas. Lack of time for reading and evaluating scientific papers in favour of direct patient work may be one explanation. To determine the drug of choice on health economic bases may be an additional difficulty. Indeed, only a minority of doctors have been educated about drug costs [1]. Therefore, it is essential to provide doctors with producer-independent information, including cost-effectiveness, to facilitate choice of treatment. Cochrane reviews indicate that audit and feedback [2] as well as interactive workshops [3] and educational outreach visits [4] can be effective in improving professional practice. However, written information may be an alternative, and adherence has been demonstrated both to individualized (patient-specific) recommendations [5] and general written information [6].

In the region of Västra Götaland in Sweden, prescribing guidelines, drawn up by 24 expert groups and determined by the regional board for drugs, are since 2006 available in the form of an annually published booklet. The expert groups consist of one chairman, one clinical pharmacologist (in some cases these positions are held by the same person), one secretary (pharmacist) and 5–10 specialist doctors with knowledge and interest in drugs in their particular therapeutic area. The prescribing guidelines include lists of recommended drugs for common diagnoses based on evidence regarding safety, cost-effectiveness and clinical experience, as well as therapy advice on how to manage the different conditions. The prescribing guidelines are assembled into a booklet, which is updated every year and distributed to all doctors in the region. The guidelines are also available on the Internet.

About 1,000 primary health care doctors work in the region of Västra Götaland. Like in the rest of Sweden, most primary health care doctors work in publicly run units, whereas a minority work in privately run units or have a business of their own. Publicly run units always bear the expenses for their prescribed drugs. Private units, on the other hand, have varying agreements concerning expenses for prescribed drugs, and are only occasionally responsible for their drug costs.

To the best of our knowledge, no information concerning how the prescribing guidelines booklet is used and attitudes towards it is available. Attitudes towards, and the impact of, committees providing prescribing advice have been investigated previously in Sweden, but data have been published only in the form of reports written in Swedish. As for the international perspective, investigations have revealed that a majority of doctors agreed to several positive statements regarding prescribing guidelines, but at the same time a substantial minority agreed to a number of negative statements (see Discussion) [7,8]. The aim of the present study was to investigate use of and attitudes towards the prescribing guidelines booklet in primary health care doctors, and to investigate if these vary according to type of organization, competence level or gender.

## Methods

All primary health care units in the region of Västra Götaland in Sweden were included in the present questionnaire study. No exact number of doctors working in the primary health care is registered. Based on available information, the number of doctors was estimated to about 1,000. Questionnaires (Additional file 1) were sent to the secretaries of multi-doctor-units (n = 154), to be administered to the doctors working at the unit. In the one-doctor units (n = 99), the questionnaires were addressed directly to the doctors in question. The questionnaires were sent at the beginning of May 2007. The doctors were instructed to fill in the questionnaire anonymously and to return it in a pre-addressed envelope. One email reminder, expected to reach most doctors in publicly run health care units, was sent at the beginning of June. Questionnaires returned before 21 June 2007 were included in the analyses.

The questionnaire consisted of 16 questions, concerning (i) characteristics of the responder: gender, year of registration, level of doctor competence, and type of organization of the place of work (publicly run, privately run or business of one's own), (ii) use of the prescribing guidelines booklet, including self-estimated percentage adherence to the lists of recommended drugs (i.e. percent of prescribing occasions at which the responder wittingly use these lists) as well as self-estimated use of the therapy advice sections, and (iii) attitudes towards the prescribing guidelines booklet. The latter questions were constructed as statements to which the responder should grade his level of agreement from 1 (totally disagreement) to 6 (totally agreement). An English translation of the questionnaire is available as an appendix.

### Statistics

Statistical analyses were conducted using SPSS 14.0. Kruskal-Wallis test was used for comparisons between groups. To facilitate the interpretation of the results on the attitude questions for the reader, arithmetic mean values are shown in addition to median values, even though the results are not expected to be normally distributed. A P-value < 0.05 was considered significant. Percentages are based on the total number of responders to the particular question.

## Results

Totally 603 doctors returned a filled-in questionnaire; estimated response rate 60% (see Methods section). The same response rate was estimated for doctors at publicly and privately run multi-doctor units, whereas only about 32% of doctors running their own business responded to the questionnaire.

Characteristics of the doctors are described in table 1. Median (25^th ^– 75^th ^percentile) year of registration was 1989 (1981–2000). In the subgroups doctors of publicly run units, doctors of privately run units and doctors running their own business, median year of registration was 1990, 1989 and 1980, respectively; specialists accounted for 67%, 87% and 100%, respectively; and males for 50%, 67% and 81%, respectively.

**Table 1:** Characteristics of doctors who returned a filled-in questionnaire.

		n (%)
Male/female		321/277 (54/46)

Type of organization	Publicly run multi-doctor-unit	486 (82)
	Privately run multi-doctor-unit	76 (13)
	Business of one's own	32 (5)

Level of doctor competence	Specialist in primary health care	430 (72)
	Resident	100 (17)
	Intern	30 (5)
	Any other competence	40 (7)

Totally 571 doctors (97%) responded that they use the prescribing guidelines booklet, whereas 14 (2%) knew about its existence but do not use it. Six doctors (1%) did not recognize the existence of the prescribing guidelines booklet.

For all doctors, self-estimated adherence to the prescribing guidelines of the booklet was in median (25^th ^– 75^th ^percentile) 80 (75–90)% when prescribing drugs for a new diagnosis and 50 (20–70)% as for active changes to a recommended drug upon renewal of a prescription. Results in subgroups of doctors are presented in table 2.

**Table 2:** Use of the drug recommendations varies with both type of organization and level of competence.

	Prescribing of a drug for a new diagnosis	P-value*	Active change to a recommended drug upon renewal of a prescription	P-value*
Publicly run units	80 (76–90)		50 (20–70)	
Privately run units	80 (75–90)		50 (20–79)	
Own business	65 (50–80)	0.0003	22 (11–50)	0.048

Specialists	80 (75–90)		50 (25–75)	
Residents	80 (72–90)		30 (10–50)	
Interns	80 (55–90)	0.81	10 (0–28)	10^-10^

*Kruskal-Wallis test for comparison between groups

Self-estimated percentage values in subgroups of doctors, as for how often (percent of prescribing occasions) the responder wittingly adheres to the lists of recommended drugs in the prescribing guidelines booklet. Values are presented as median percentage (25^th ^– 75^th ^percentile).

**Figure 1:**
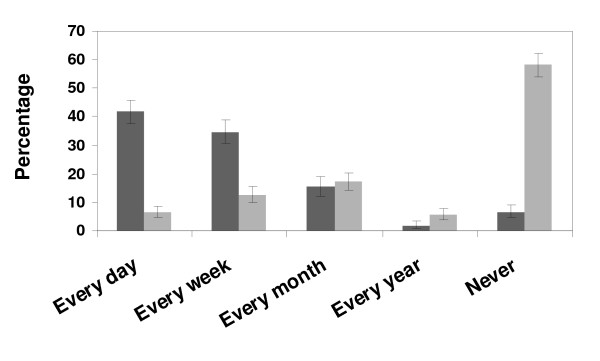
**The therapy advice in the booklet is frequently used, in contrast to the Internet-based therapy advice**. Use of the therapy advice sections of the prescribing guidelines in the booklet (dark grey bars) and on the Internet (light grey bars). Error bars denote 95% confidence intervals.

The use of the therapy advice sections of the prescribing guidelines in the booklet and on the Internet is described in figure 1. Among the 231 doctors (42%) using the therapy advice section in the booklet every day, 47 doctors (8% of all) use it ≥ 5 times daily; and of the 40 doctors (7%) using the therapy advice on the Internet, 12 doctors (2% of all) use it ≥ 5 times daily.

**Figure 2:**
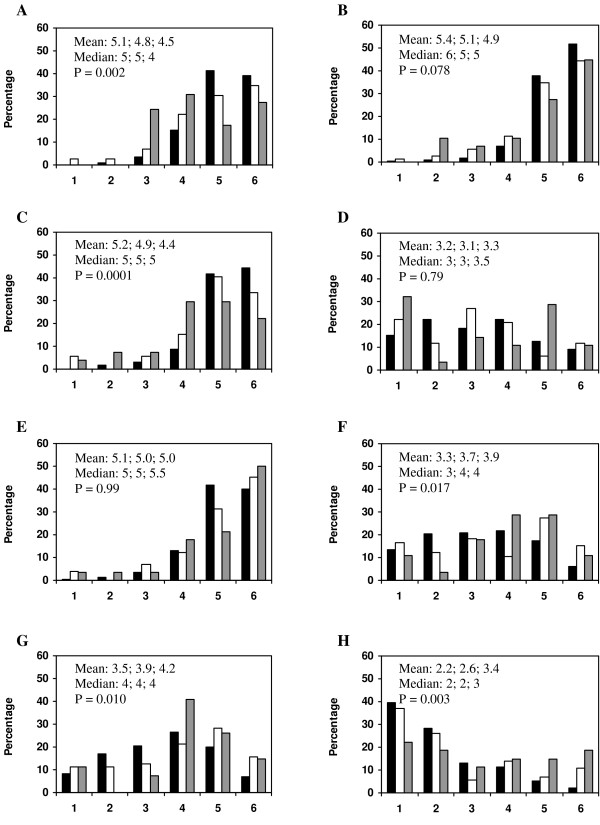
**Doctors' attitudes towards the prescribing guidelines booklet vary between different types of organizations**. Doctors' agreement in the statements: (A) I trust the recommended drug list to reflect sound judgments concerning effects and safety, (B) I trust the recommended drug list to reflect sound judgments concerning cost-effectiveness, (C) When I adhere to the recommended drug list, I do it to attain evidence-based prescribing concerning effects and safety, (D) When I adhere to the recommended drug list, I do it because it is required by the health care system, (E) When I adhere to the recommended drug list, I do it to attain sound health economics in the community. (F) I refrain from changing a not recommended drug to a recommended one due to experience of misuse of the drug by the patient (due to the patient's misunderstanding), (G) I refrain from changing a not recommended drug to a recommended one due to the risk of misuse of the drug by the patient (due to the patient's misunderstanding), (H) The prescribing guidelines, and activities aiming at visualizing adherence to it (such as outreach visits of pharmacists presenting prescribing statistics for the particular primary health care unit), trespass the freedom of the profession. The responder was to grade his level of agreement from 1 (totally disagreement) to 6 (totally agreement). Black bar denotes publicly run unit, white bar denotes privately run unit and grey bar denotes business of one's own. Arithmetic mean and median, respectively, are presented in the following order throughout the figure: publicly run unit; privately run unit; business of one's own. Kruskal-Wallis test was used for comparisons between groups.

Attitudes towards the prescribing guidelines booklet among the subgroups (i) doctors working at publicly run primary health care units, (ii) doctors working at privately run units and (iii) doctors having their own business are described in figure 2.

To investigate if demographic differences (years after registration, competence level, gender) between doctors of publicly run units, doctors of privately run units and doctors running their own business could account for the above differences, a matched group of doctors from publicly run units (fulfilling the criteria year of registration 1960–1995, specialists only, men only; n = 144) was compared to male doctors running their own business (n = 25). This comparison showed differences almost identical to those between publicly run units and businesses of one's own presented above (data not shown), suggesting that these differences are not due to demographic confounders.

Male interns currently working in primary health care judged the prescribing guidelines, and activities aiming at visualizing adherence to it, to trespass the freedom of the profession to a higher degree than their female equivalents [mean: 3.0 and 1.9, median: 3 and 2, respectively, P = 0.028]. In contrast, no gender difference was found in residents or specialists in primary health care medicine.

**Figure 3:**
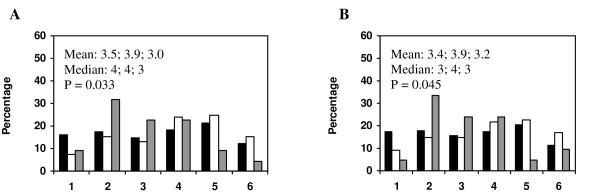
**Attitude questions concerning extra work are ranked differently by specialists, residents and interns**. Doctors' agreement in the statements: (A) I refrain from changing a not recommended drug to a recommended one due to experience of extra work, and (B) I refrain from changing a not recommended drug to a recommended one due to the risk of extra work. The responder was to grade his level of agreement from 1 (totally disagreement) to 6 (totally agreement). Black bar denotes specialists, white bar denotes residents and grey bar denotes interns. Arithmetic mean and median, respectively, are presented in the following order throughout the figure: specialists; residents; interns. Kruskal-Wallis test was used for comparisons between groups.

Attitudes towards the prescribing guidelines booklet among the subgroups (i) specialists, (ii) residents and (iii) interns differed concerning reasons for changing a not-recommended drug to a recommended one. The results are presented in figure 3.

## Discussion

The results of the present study indicate that the prescribing guidelines booklet is frequently used and adhered to by primary health care doctors. It was also deemed to reflect sound judgements concerning effects, safety and cost-effectiveness. The effort to draw up these guidelines and to administer them in the format of a booklet hence seems worthwhile. Access to producer-independent information is an important counterweight to drug information from drug industry representatives, since frequent visits by these have been shown to be associated with increased prescribing costs [9,10], but no data on improved quality of health care is available as far as we know.

The overall positive attitude towards prescribing guidelines found in this study is in agreement with the results of previous investigations. A systematic review of thirty studies revealed that about 70% of clinicians considered guidelines helpful sources of advice, good educational tools and intended to improve quality [7]. In another study not included in that review, a similar percentage of responding general practitioners believed that well-constructed guidelines were effective in improving patient care [8].

The prescribing guidelines were generally not experienced to trespass the freedom of the profession; 22% of responders graded their level of agreement to ≥ 4 out of 6. This could be in accordance with a previous study, where 25% feared that guidelines can diminish clinical freedom and stifle innovation [8]. In the above mentioned review, a substantial minority considered guidelines impractical and too rigid to apply to individual patients (30%), and to reduce physician autonomy and oversimplify medicine (34%) [7]. As for the freedom of the profession, several doctors in the present study spontaneously wrote that it is crucial that the prescribing guidelines remain recommendations, as opposed to compulsive instructions.

No unambiguous reason for not changing a not recommended drug to a recommended one could be detected. Doctors in publicly run primary health care units were generally more positive to the prescribing guidelines and adhered to them more often. This is in accordance with previous data available only in Swedish. Financial incentives may be one explanation for this finding, as privately run units in Sweden more seldom are responsible for their prescribed drug costs. Indeed, an association between budgetary policies and prescribing performance has been shown [11,12].

Specialists in primary health care were more prone to actively change from a not recommended drug to a recommended one upon renewal of a prescription, when compared with residents and interns. An augmented comprehension of the difficulties in prescribing and appreciation of guidelines by age and experience may be one explanation for this finding. Another may be that less experienced doctors find it difficult to change prescriptions made by senior colleagues. Moreover, they may feel more stressed in the working situation, which could make them less tolerant to time-consuming activities that they do not find totally necessary. Indeed, residents avoided active changes to a recommended drug, based on the experience and potential risk of extra work, more often than specialists. Interns, however, ranked these as less important motives for avoiding active changes to a recommended drug, which could reflect their relative lack of experience, and that collegiate reasons may overshadow the risk of extra work in this group. Notably, less experienced doctors did not use the prescribing guidelines less frequently than more experienced colleagues when prescribing a drug for a new diagnosis, a situation in which recommendations could be time-saving rather than time-consuming.

Gender differences as for attitudes to the prescribing guidelines booklet were seen only in interns, where male interns found it to trespass the freedom of the profession to a greater degree than female equivalents. This may be of principal interest, as interns were the only responders in the present study who have not decided to make a career in primary health care. (In Sweden, internship is required to get a general medical license and part of the internship must be fulfilled in primary health care.) Thus, it would be of interest to investigate if attitudes towards prescribing guidelines differ between doctors of different medical specialities (broad and narrow ones, traditionally male and female ones, et cetera) and between genders within different specialities. Such studies are now planned by the undersigned.

Prescribing guidelines in the booklet format seem to be used more frequently than Internet-based information. This may illustrate the importance of easy accessibility in the stressful clinical situation. Nonetheless, computer assistance has been shown to improve drug dosage [13] and computerized prescribing assistance is a question of importance.

This study has several limitations. Firstly, the response rate was around 60%, yielding a possibility of substantial selection bias, where, possibly, individuals negative to the guidelines could have been less prone to respond. Doctors running their own business showed an even lower response rate, 32%, and may therefore be even more negative to the guidelines than indicated by the results of this study. Non-response could not be coupled to other demographic or geographic parameters, since the responders were anonymous. Secondly, the present study only allows conclusions regarding doctors' estimates on the prescribing guidelines and their adherence to it. Actual effects of the guidelines on prescribing patterns should be further explored, and such studies are planned by the authors. Thirdly, multiple analyses and several subgroup analyses have been performed in this study. This increases the probability of making type I errors (false differences between groups), especially in small subgroups. Analyses of small subgroups, such as the gender analysis of interns, should only be seen as hypothesis generating.

Regarding practical implications of this study for future prescribing guidelines work, the results may indicate a need for increased user-friendliness as for the Internet based guidelines, and need for intensified marketing of the guidelines booklet towards doctors working outside the public health care system.

## Conclusion

The prescribing guidelines were judged to be frequently used and are generally appreciated, though differences exist between subgroups of users.

## Competing interests

MABA, AM and SMW are involved in the expert groups drawing up the prescribing guidelines. AM is editor of the prescribing guidelines booklet.

## Authors' contributions

MABA conceived the study, drafted the questionnaire, carried out acquisition and analysis of data and revised the manuscript. MS carried out acquisition and analysis of data. AM revised the manuscript. SMW performed statistical analyses and drafted the manuscript. All authors contributed to the design of the study, and read and approved the final manuscript.

## Pre-publication history

The pre-publication history for this paper can be accessed here:



## Supplementary Material

Additional file 1 **Questionnaire:** Questionnaire concerning use of and attitudes towards the prescribing guidelines booklet in primary health care doctors in the region of Västra Götaland. Click here for file
