# Supplementary material for: Use of and attitudes towards the prescribing guidelines booklet in primary health care doctors
Source: BMC Clin Pharmacol. 2008 Sep 22;8:8. doi: 10.1186/1472-6904-8-8 (PMC2556993; doi:10.1186/1472-6904-8-8)
Supplement: Additional file 1 — Questionnaire: Questionnaire concerning use of and attitudes towards the prescribing guidelines booklet in primary health care doctors in the region of Västra Götaland. [file 1472-6904-8-8-S1.doc]

Göteborg May 2007

Questionnaire

concerning use of and attitudes towards the prescribing guidelines booklet

in primary health care doctors in the region of Västra Götaland

**Dear colleague,**

The regional prescribing guidelines booklet, which contains lists of recommended drugs as well as therapy advice, has been available since 2006. We are interested in investigating use of and attitudes towards this booklet in primary health care doctors in the region, and would therefore appreciate your filling in this anonymous questionnaire and returning it in the enclosed envelope, preferably before June 8. Individual information will be available for the undersigned only. If you have questions, please contact Magnus Axelsson, telephone +46 31 342 45 17, email: magnus.axelsson@vgregion.se. The investigation *does not* include the prescribing guidelines booklet for hospitals or the list for interchangeable drugs. **Thank you for your participation!**

| Malin Spetz  MD, intern | Susanna Wallerstedt  MD, PhD | Anders Mellén  MD | Magnus Axelsson  MD, resident, PhD |
| --- | --- | --- | --- |

*All employed at the Department of Clinical Pharmacology, Sahlgrenska University Hospital*

**1. I am a/an (several alternatives can be chosen)**

□ specialist in primary health care. □ deputizing doctor in a primary health care unit.

□ resident in primary health care. □ rented doctor in a primary health care unit.

□ intern in a primary health care unit. □ other doctor:..........................................................

**2. I am** □ female □ male. **3. Year of registration (license):** …………..

**4. I work** □ in a publicly run primary health care unit.

□ in a privately run primary health care unit. □ in a business of my own.

**5. I** □ do not know of the prescribing guidelines booklet (leave the rest of the questionnaire unanswered and return the questionnaire to us!) □ know of the prescribing guidelines booklet, but I do not use it □ use the prescribing guidelines booklet

**6. My prescribing guidelines booklet for 2007 arrived at the correct address in due time (February 1).**

□ Yes □ No, it arrived ……… □ No, I have not received one.

**7. When prescribing a drug for a new diagnosis, I wittingly adhere to the lists of recommended drugs in about ………. % of prescribing occasions**.

**8. Upon renewal of a prescription, I actively change to a recommended drug in about ………. % of prescribing occasions.**

**9. I wittingly use/adhere to the therapy advice in the prescribing guidelines booklet about ………. times** □ every day / □ every week / □ every month / □ every year. □ Never.

**10. I wittingly use/adhere to the therapy advice on the Internet about ………. times**

□ every day / □ every week / □ every month / □ every year. □ Never.

**11. Taking quality and resources into account, prescribing guidelines should be produced**

□ at a regional level (like at present).

□ at a still more local level (e.g. like previously, with five local booklets).

□ at a national level.

| **1.**  **I totally disagree** | **6.**  **I totally agree** | **No opinion** |
| --- | --- | --- |

| **12. I trust the recommended drug list to reflect sound judgments concerning**  a) effects and safety.  b) cost-effectiveness. | 1 □ 2 □ 3 □ 4 □ 5 □ 6 □ □  1 □ 2 □ 3 □ 4 □ 5 □ 6 □ □ |
| --- | --- |
| **13. When I adhere to the recommended drug list, I do it**  a) because it is required by the health care system.  b) to attain sound health economics in the community.  c) to attain evidence-based prescribing concerning effects and safety. | 1 □ 2 □ 3 □ 4 □ 5 □ 6 □ □  1 □ 2 □ 3 □ 4 □ 5 □ 6 □ □  1 □ 2 □ 3 □ 4 □ 5 □ 6 □ □ |
| **14. I refrain from changing a not recommended drug to a recommended one due to**  a) experience of misuse of the drug by the patient (due to the patient’s misunderstanding).  b) the risk of misuse of the drug by the patient (due to the patient’s misunderstanding).  c) experience of extra work.  d) risk of extra work. | 1 □ 2 □ 3 □ 4 □ 5 □ 6 □ □  1 □ 2 □ 3 □ 4 □ 5 □ 6 □ □  1 □ 2 □ 3 □ 4 □ 5 □ 6 □ □  1 □ 2 □ 3 □ 4 □ 5 □ 6 □ □ |
| **15. The prescribing guidelines, and activities aiming at visualizing adherence to it (such as outreach visits of pharmacists presenting prescribing statistics for the particular primary health care unit), trespass the freedom of the profession.** | 1 □ 2 □ 3 □ 4 □ 5 □ 6 □ □ |
| **16. When a new edition of the prescribing guidelines booklet appears, I bring myself up to date concerning revisions.** | 1 □ 2 □ 3 □ 4 □ 5 □ 6 □ □ |

**Comments:………………………………………………………………………………….**

**..……………………………………………………………….……………………………..**

**..……………………………………………………………….……………………………..**
